# Preoperative Tyrosine Levels as Predictive Biomarkers for Excessive Fat-Free Mass Loss Following Laparoscopic Sleeve Gastrectomy in Patients with Morbid Obesity

**DOI:** 10.3390/metabo15080543

**Published:** 2025-08-11

**Authors:** Inyoung Lee, Eunhye Seo, Yeongkeun Kwon, Chang Min Lee, Nam Hoon Kim, Jong-Han Kim, Sung Il Choi, Sungsoo Park

**Affiliations:** 1 Division of Foregut Surgery, Department of Surgery, Korea University College of Medicine, Seoul 02841, Republic of Korea; irene.inyoung.lee@gmail.com (I.L.); kwon.yeongkeun@gmail.com (Y.K.); laparosamurai@gmail.com (C.M.L.); ppongttai@gmail.com (J.-H.K.); 2 College of Nursing, Keimyung University, Daegu 42601, Republic of Korea; ehseo@kmu.ac.kr; 3 Division of Endocrinology and Metabolism, Department of Internal Medicine, Korea University College of Medicine, Seoul 02841, Republic of Korea; pourlife@korea.ac.kr; 4 Department of Surgery, Kyung Hee University Hospital at Gangdong, Seoul 05278, Republic of Korea; drchoi@khu.ac.kr

**Keywords:** metabolic and bariatric surgery, tyrosine, amino acid metabolites, aromatic amino acids

## Abstract

**Background/Objectives**: Fat-free mass (FFM) loss after metabolic and bariatric surgery (MBS) is associated with adverse long-term outcomes, including osteoporosis. Identifying biomarkers that predict excessive FFM loss can improve perioperative patient management and postoperative risk stratification. This study investigated whether preoperative amino acid metabolite (AAM) levels could predict excessive FFM loss after laparoscopic sleeve gastrectomy (LSG). **Methods**: Forty patients with morbid obesity who underwent LSG between 2019 and 2020 were retrospectively analyzed. Based on the FFM loss to body weight loss ratio (%FFML/BWL) at 3 months postoperatively, patients were categorized into excessive (>25%) and non-excessive (≤25%) FFM loss groups. Anthropometric measurements and serum sampling were performed preoperatively and at 3, 6, and 12 months postoperatively. AAM profiles were collected before surgery. Statistical analyses, including logistic regression and receiver operating characteristic curves, were performed. **Results**: Twenty-five patients showed excessive FFM loss 3 months after surgery. They had significantly lower preoperative tyrosine (Tyr) levels (*p* = 0.025). Logistic regression revealed that higher Tyr levels were significantly associated with lower odds of being male, suggesting a potential protective effect (odds ratio (OR) =0.019, *p* = 0.010). Tyr profiling demonstrated acceptable predictive performance (area under the curve =0.715, *p* = 0.025). Despite nonsignificant *p*-values, trends showed lower FFM and muscle mass and higher fat mass in the excessive FFM loss group throughout follow-up. **Conclusions**: Preoperative Tyr profiling may help identify patients at risk for excessive FFM loss. These findings support prioritizing metabolic health alongside total weight loss in the evaluation of MBS outcomes.

## 1. Introduction

**Figure 1:**
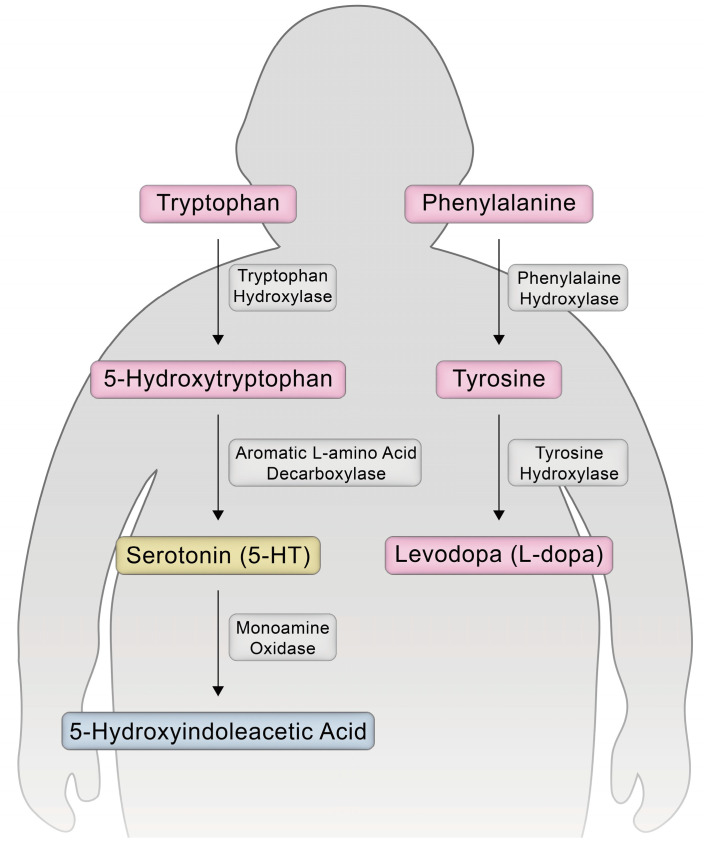
Metabolic pathways for tryptophan and phenylalanine.

Understanding the metabolic mechanisms underlying obesity has become a research priority as metabolomics has proven to be a powerful tool for identifying key biomarkers and elucidating their mechanisms. Key molecular players associated with insulin resistance, body composition changes, and metabolic responses after metabolic and bariatric surgery (MBS) include the aromatic amino acid (AAA) group of amino acid metabolites (AAMs) (Figure 1) [1]. Phenylalanine (Phe), tyrosine (Tyr), and tryptophan (Trp) are associated with appetite suppression and glucose control [2,3,4,5,6]. In the gut microbiome, Trp is further metabolized into serotonin (5-HT), a neurotransmitter that is increased in patients with obesity and is related to glucose control [7,8,9]. A metabolite of 5-HT, 5-Hydroxyindoleacetic acid (5-HIAA), affects metabolic and glycemic homeostasis. Phe and Tyr levels increase in patients with obesity and type 2 diabetes mellitus (T2DM) [6,9,10,11]. Thus, circulating levels of AAMs are altered in individuals with obesity and insulin resistance and are closely linked to glucose metabolism and inflammatory pathways [2,3,4,5,6,9,10]. Changes in their profiles have been associated with metabolic responses to MBS [3,6,9]. Therefore, the preoperative profiling of AAMs may serve as an indicator of treatment response or metabolic changes.

In patients with morbid obesity, MBS induces significant weight loss and alleviates obesity-related diseases. Laparoscopic sleeve gastrectomy (LSG) is the most frequently performed procedure worldwide and offers effective weight reduction, T2DM improvement, and a relatively low complication rate [12]. In East Asian countries, where the incidence of gastric cancer is high, the advantages include preservation of endoscopic access for future cancer surveillance [13,14]. Body weight (BW) reduction leads to substantial changes in body composition. BW consists of fat mass (FM) and fat-free mass (FFM, also known as lean mass), which includes skeletal muscles, bones, body fluids, and organs [15]. Post-MBS patients experience a reduction in both FM and FFM, with maximum BW reduction at 3 months [16,17,18]. The early postoperative phase accounts for 50% of total FFM loss [15]. The FFM loss relative to body weight loss (%FFML/BWL) was >20% in most procedures, with an average of 24.8% in post-MBS patients [15]. The “Quarter FFM rule”, which is based on several literature reviews and cohort studies, identifies 25% as the physiological threshold of %FFML/BWL that distinguishes adaptive weight loss from potentially excessive lean mass depletion [15,19]. When excessive FFM loss occurs, the reduction in muscle and bone mass increases the long-term risk of osteoporosis, sarcopenia, fat mass accumulation, and weight regain [19,20,21,22,23,24,25]. We aimed to evaluate whether AAMs can serve as early biomarkers for identifying patients susceptible to excessive lean mass loss after MBS, thereby supporting risk stratification and personalized postoperative management. We hypothesized that distinct preoperative profiles of AAMs may serve as early predictors of excessive FFM loss at 3 months after laparoscopic sleeve gastrectomy.

## 2. Materials and Methods

### 2.1. Study Participants

We retrospectively analyzed 40 patients with obesity who underwent LSG performed by a single expert surgeon between January 2019 and December 2020 at the Center for Obesity and Metabolic Diseases, Korea University Anam Hospital, Seoul, South Korea. Eligible patients were >18 years of age with a BMI ≥ 35 kg/m^2^ or a BMI ≥ 30 kg/m^2^ with obesity-related comorbidities and had failed to lose weight using non-surgical methods. Patients with a history of MBS, complex abdominal surgery, or uncontrolled medical or psychiatric conditions were excluded. Given the retrospective nature and limited sample size (*n* = 40), no formal power calculations were conducted.

### 2.2. Data Collection

Data were collected preoperatively and at 3-, 6-, and 12-month postoperative follow-up visits to the outpatient clinic, including anthropometric evaluations, serum sampling, and body composition analyses. Body composition data were assessed using bioimpedance analysis (BIA) with a Quad Scan 4000 multi-frequency bioelectrical impedance analyzer (Body Stat^®^, Isle of Man, UK), using impedance at 50 kHz and applying the corresponding equations to measure FM, muscle mass (MM), and FFM. All 40 patients completed the 3- and 6-month follow-up visits. At 12 months postoperatively, 38 patients attended the scheduled clinic visit; however, only 32 completed the BIA examination.

AAM profiling and analyses were performed on the serum samples collected preoperatively. Although 6-month measurements were available for the subset, they were excluded from the analysis owing to the lack of statistical significance and the focus on identifying preoperative biomarkers predictive of postoperative FFM reduction. Seven metabolites were included based on their known associations with obesity-related metabolic regulation as previously described: Phe, Trp, Tyr, 5-HT, 5-HTP, 5-HIAA, and levodopa [1,2,3,4,5,6,7,8,10]. Metabolite profiling was performed using liquid chromatography/tandem mass spectrometry (ABSciex 3200 High-performance liquid chromatography–mass spectrometry/mass spectrometry with 1200 Agilent HPLC; Agilent Technologies, Palo Alto, CA, USA) using a Waters Atlantis T3 column, as previously described by our group [1,26]. Serum samples were processed by protein precipitation using acetonitrile, followed by chromatographic separation and detection via multiple reaction monitoring in positive ionization mode. Internal standard 13^C^-Trp (5 μM) was used to ensure quantification accuracy, and data analysis was performed using the Analyst software (Version 1.6.3, SCIEX, Framingham, MA, USA) [1,26].

### 2.3. Patient Classification and Outcome Measurements

According to previous studies on MBS, maximum weight loss typically occurs within the first 3 months after surgery, during which approximately 50% of the total FFM reduction is observed [16,17,18]. Therefore, this period is considered a critical phase for early postoperative changes in body composition [19]. Moreover, the %FFML/BWL observed after LSG was approximately 25% (24.8%) [15]. Based on evidence suggesting that a %FFML/BWL ≈ 25% represents an optimal level of weight loss in patients with obesity, patients were classified into two groups accordingly [19,20,21,22,23,24,25]. This 3-month timeframe was selected based on studies indicating that approximately half of the postoperative FFM reduction occurs during this period, making it the most metabolically informative phase for stratifying lean mass loss [14,15,16,17,18].

The %FFML/BWL was calculated as follows:

$$\%\frac{FFML}{BWL}=\frac{{FFM}_{postoperative}-{FFM}_{preoperative}}{{BW}_{postoperative}-{BW}_{preoperative}}\times 100\%$$

Patients were classified based on %FFML/BWL at 3 months postoperatively. Those with a %FFML/BWL ≤ 25% were assigned to the control group, and those with a %FFML/BWL > 25% were classified into the “Excessive Fat-Free Mass Loss” group.

Weight loss outcomes were calculated using the equations for % total weight loss (%TWL) and % excess weight loss (%EWL) using ideal BW calculations with Devine’s formula [27].

### 2.4. Statistical Analysis

Statistical analyses were conducted using SPSS software (version 23.0; IBM SPSS Inc., Armonk, NY, USA). The normality of variables was confirmed using the Shapiro–Wilk test. The chi-square test was used for the analysis of categorical variables, and the independent Student’s t-test or Mann–Whitney U test was used for group comparisons, depending on the distribution of the data. Statistical significance was defined as a *p*-value < 0.05, with a significance level of 5% (95% confidence interval) and statistical power of 80%. A logistic regression model was used to analyze the relationship between serum AAMs and %FFML/BWL 3 months postoperatively. The model was adjusted for baseline variables, including sex, age, BMI, and FFM, based on clinical relevance and statistical considerations. Specifically, sex was included because of its significant between-group difference in the primary outcome comparison of preoperative characteristics between the two groups, and age was included because of its well-established relationship with FFM decline over time [28]. Preoperative BMI and FFM were incorporated to control baseline body composition and anthropometric variations, both of which could confound the predictive value of metabolic biomarkers for postoperative body composition outcomes. Model fit was compared using the −2 Log Likelihood (−2LL) statistic, where lower values indicate a better fit. Model calibration was assessed using the Hosmer–Lemeshow (HL) test, where a nonsignificant result (*p* > 0.05) indicates good agreement between predicted and observed outcomes. Differences between models were used to assess improvements in predictive ability. To evaluate the predictive ability of the variables for excessive %FFML/BWL at 3 months postoperatively, receiver operating characteristic (ROC) curves and the area under the curve (AUC) were employed. To evaluate the discriminative ability of individual variables, ROC curve analysis was performed for both multivariable logistic regression models and single-variable models. To complement non-parametric analyses, the effect size (r) for each Mann–Whitney U and Wilcoxon signed-rank test was determined based on the Z-statistic divided by √N, where N is the number of paired or pooled observations. Interpretation followed Cohen’s thresholds: r ≥ 0.5 (large), r ≥ 0.3 (moderate), and r ≥ 0.1 (small), and results are included in the Supplementary Materials. To assess the statistical sensitivity of our non-parametric tests, post hoc analysis was conducted using G*Power (version 3.1.9.7). For between-group comparisons, Mann–Whitney U and Wilcoxon signed-rank tests were performed, and the results are also included in the Supplementary Materials.

### 2.5. Ethics Approval

This study was approved by the Institutional Review Board of Korea University Anam Hospital (IRB No. 2019AN0132) and conducted in accordance with the ethical standards of the Declaration of Helsinki. The requirement for informed consent was waived due to the retrospective design.

## 3. Results

### 3.1. Preoperative Patient Demographics

Forty patients with morbid obesity underwent LSG between 2019 and 2020. The average age was 37.4 ± 11.3 years, with a higher ratio of females (female/male = 2.08:1), and 72.5% of the patients had at least one concurrent metabolic disease, such as T2DM, hypertension, or dyslipidemia (Table 1).

### 3.2. Postoperative Outcomes

Postoperative 3-, 6-, and 12-month anthropometric parameters revealed significant weight loss compared with the preoperative values (Table 2). The FFM-to-BW ratio, %TWL, and %EWL all increased significantly through postoperative month 12 (*p* < 0.001). %FFML/BWL was highest at 3 months after surgery (37.6 ± 28.8%).

### 3.3. Outcomes for the Excessive FFM Loss Group

Preoperative characteristics of the two groups (control group with %FFML/BWL ≤ 25%, and Excessive FFM Loss group with %FFML/BWL > 25%) showed a higher ratio of females in the Excessive FFM Loss group than the control group (Table 3).

Weight loss outcomes (%TWL, %EWL) did not differ significantly between groups (Table 4). The Excessive FFM Loss group showed a consistently increased %FFML/BWL for up to 12 months postoperatively.

**Figure 2:**
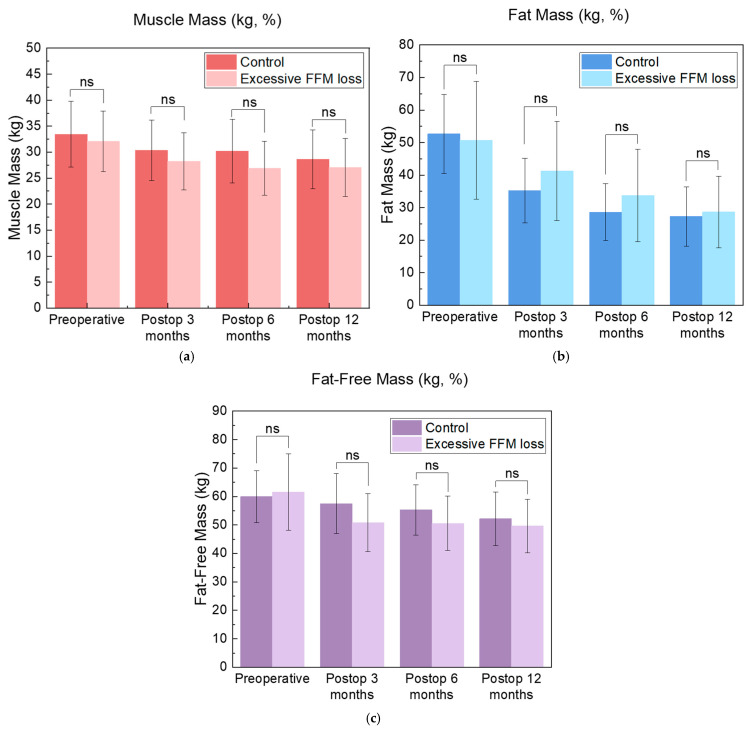
MM (**a**), FM (**b**), and FFM (**c**) values preoperatively and postoperatively at 3, 6, and 12 months. “ns” indicates no significant difference. Abbreviations: Postop, postoperative; FFM, fat-free mass.

Although not statistically significant, the control group exhibited increased MM and FFM and decreased FM (Figure 2). Subgroup analyses of postoperative clinical laboratory data were omitted owing to the insufficient sample size from loss to follow-up, limiting statistical interpretability.

When the AAM values were compared between the two groups, Trp (*p* = 0.015) and Tyr (*p* = 0.025) were lower in the Excessive FFM Loss group (Table 5).

Unadjusted outcomes for excessive FFM loss (%FFML/BWL > 25% at postoperative 3 months) prediction using logistic regression analyses showed that both Trp and Tyr reached statistical significance (Trp: OR = 0.919, 95% CI = 0.850–0.993, *p* = 0.033; Tyr: OR = 0.932, 95% CI = 0.877–0.990, *p* = 0.022), with Tyr demonstrating superior model fit (Nagelkerke R^2^ = 0.528; Hosmer–Lemeshow (HL) *p* = 0.920) (Table 6). When adjusted for age, BMI, and FFM, both AAMs remained statistically significant (Trp: OR = 0.914, *p* = 0.027; Tyr: OR = 0.927, *p* = 0.018), although the model fit improved with Tyr (Nagelkerke R^2^ = 0.258 vs. 0.229). The inclusion of sex in the final model (Model 3) further strengthened the predictive utility of Tyr (OR = 0.901, 95% CI = 0.830–0.977, *p* = 0.012), and male sex emerged as a significant protective factor (OR = 0.018, 95% CI = 0.001–0.359, *p* = 0.008). This comprehensive analysis indicates that Tyr is the most robust metabolic predictor of early excessive FFM loss, particularly when combined with demographic and body composition variables. Multicollinearity was assessed for the predictors evaluated in the logistic regression analyses, and all variables demonstrated variance inflation factor values < 5 and tolerance > 0.1, indicating no serious multicollinearity issues (Supplementary Table S6).

**Figure 3:**
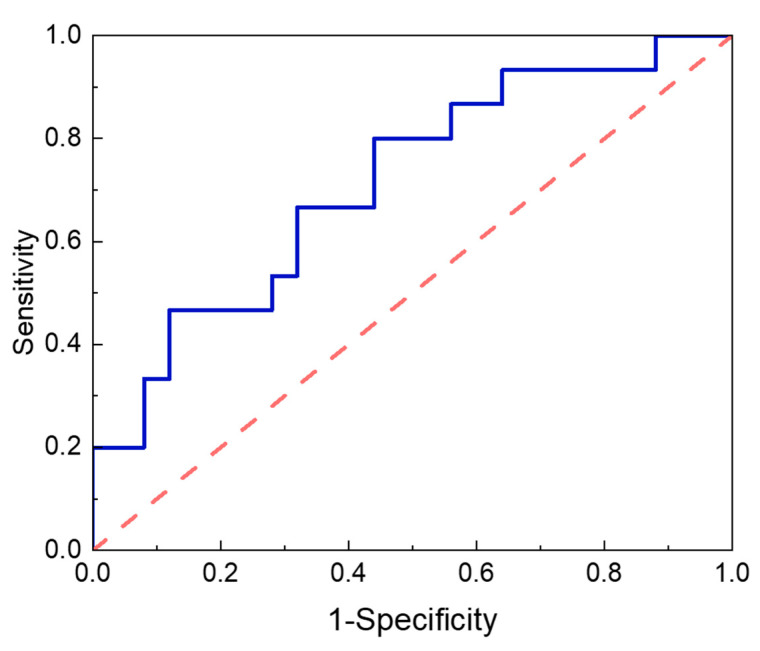
Predicting excessive FFM loss using ROC curve analysis of preoperative Tyr levels. Abbreviations: %FFML/BWL, percentage of fat-free mass loss relative to total body weight loss; FFM, fat-free mass; ROC, receiver operating characteristics.

To evaluate and compare the discriminative performance of the predictive variables and models, ROC analysis was performed using the predicted probabilities generated using logistic regression. When analyzed as a single-variable model, Tyr demonstrated acceptable discriminative performance (AUC = 0.715; 95% CI, 0.550–0.880; *p* = 0.025) in predicting excessive FFM loss (Figure 3) (Table 7). For comparison, male sex yielded a moderate but nonsignificant AUC of 0.667 (95% CI, 0.486–0.847; *p* = 0.081), whereas preoperative FFM displayed poor predictive performance as a standalone marker (AUC = 0.519; 95% CI, 0.338–0.699; *p* = 0.845). ROC curves for variables other than Tyr are not shown as they did not demonstrate statistical significance.

## 4. Discussion

This study highlights the potential of preoperative profiling of amino acid metabolites, particularly Tyr, as early identifiers of patients at risk for excessive FFM loss after MBS. Given that FFM loss is associated with long-term adverse outcomes, these findings offer a foundation for risk stratification and targeted interventions. We examined whether preoperative AAM profiles could predict body composition changes in post-LSG patients using FFM loss as a qualitative indicator. Patients with a %FFML/BWL > 25%, a threshold supported by previous systematic reviews and meta-analyses, were classified as having an excessive mass loss [15]. These patients exhibited significantly lower preoperative Tyr profiles, and logistic regression and ROC analyses confirmed Tyr as a significant predictor of excessive FFM loss at postoperative 3 months (AUC = 0.715, *p* = 0.025). Male sex also appeared to be a protective factor against excessive lean mass loss (OR = 0.022, *p* = 0.047); however, the AUC was not statistically significant (AUC = 0.667, *p* = 0.081), suggesting that both metabolite- and sex-related factors may influence postoperative FFM loss. To our knowledge, this is the first study to identify preoperative tyrosine as a predictive biomarker of early postoperative excessive FFM loss following laparoscopic sleeve gastrectomy. While the majority of the existing literature has centered on metabolic disease improvement and fat or adipose tissue mass reduction in MBS patients, fewer studies have investigated FFM loss as a distinct clinical outcome of MBS, and even fewer have explored its association with AAMs [9,11,29]. For instance, systematic reviews have emphasized the impact of MBS on underlying metabolic diseases but have not specifically addressed lean mass loss or its association with metabolites [30,31]. Our study emphasizes the importance of monitoring early FFM reduction, which may carry long-term risks that are underappreciated in standard post-MBS assessments. This focus adds a novel layer to the existing body of research, underscoring the need for integrative models that predict and mitigate not just fat reduction but also potential adverse changes in body weight composition.

Our findings are consistent with those of a previous study that demonstrated sex-specific associations between AAMs and body composition. A study of Finnish adults reported a positive correlation between higher FFM and Tyr levels in men, whereas higher Tyr levels were associated with higher FM in women [32]. In our analysis, male sex was associated with a lower likelihood of excessive FFM loss, and the proportion of men in the Excessive FFM Loss group was significantly lower than that in the control group (male/ female = 1:4, *p* = 0.029). Additionally, preoperative Tyr level was inversely associated with early FFM loss. Together, these findings suggest that Tyr may reflect the underlying metabolic phenotypes that differ by sex and the potential to utilize preoperative AAM profiling with sex-specific reference ranges in future personalized approaches for risk stratification. However, AAM levels did not differ significantly between men and women across the entire cohort or within stratified groups in a non-parametric analysis. Although male sex emerged as a protective factor in our model, the observed sex effect may not have been driven by differences in circulating AAMs. This result may reflect broader physiological mechanisms, such as baseline lean mass differences or sex differences in hormonal levels. Therefore, the incorporation of sex into the logistic regression model was essential to isolate the independent effect of Tyr as a biomarker, which was not confounded by sex-based variance.

Notably, both unadjusted and adjusted logistic regression analyses for Trp and Tyr as predictors for excessive FFM loss at postoperative 3 months showed that both metabolites reached statistical significance (Table 6). Among the two, Tyr demonstrated superior model fit, and when sex was included as a covariate, the analysis strengthened the predictive utility of Tyr. This comprehensive analysis indicates that Tyr is the most robust metabolic predictor of early excessive FFM loss, particularly when combined with demographic and body composition variables. This observation underscores the influence of baseline clinical variables, particularly sex and preoperative body composition, on the postoperative outcome. It highlights the necessity of adjusted analyses to uncover predictive relationships and supports the utilization of Tyr as a biomarker when key covariates are considered.

Although weight loss is the primary objective of MBS, FFM reduction can lead to long-term postoperative consequences such as body weight regain, reduced metabolic efficiency, osteopenia, and sarcopenia [15,33]. Previous studies on these adverse outcomes have reported that, for every unit of FFM reduction, the basal metabolic rate decreased by approximately 1.95 kcal [34]. In addition, a higher %FFML/BWL ratio was positively associated with appetite, increasing the risk of weight regain [35]. A substantial proportion of FFM loss occurs within the first 3 months after MBS, representing a critical window for identifying patients at risk [36]. Moreover, our study focused on possible metabolite predictors of %FFML/BWL during this period, when peak FFM loss typically occurs [16,17,18]. Identifying high-risk patients during the early postoperative period could facilitate appropriate interventions such as nutritional support and exercise protocols to prevent lean mass loss.

In sarcopenic obesity (SO), muscle loss is related to increased vulnerability to frailty and a higher incidence of cardiovascular disease [37,38,39]. As illustrated by Prado, et al. [40], SO can arise from two trajectories: one characterized by progressive fat gain with concurrent muscle loss and rapid weight loss with disproportionate lean tissue depletion. A caloric deficit after MBS may induce the latter trajectory, thereby reducing both FM and FFM. Our study identified a patient subgroup with early excessive FFM loss who may follow this second course without compensatory recovery of the lean mass at later follow-up points.

The absolute values for MM and FFM in the Excessive FFM Loss group were consistently lower and those for FM were higher than those in the control group at the 3-, 6-, and 12-month postoperative follow-ups, although the difference was not statistically significant. However, the biological and clinical relevance of these trends should not be disregarded. The persistent gaps between the MM and FFM groups over time suggest a possible chronic shift in body composition, despite similar %TWL and %EWL values. This pattern may reflect chronic depletion of metabolically functioning tissues and predispose affected individuals to adverse long-term outcomes. Given the small sample size and relatively short follow-up period, the analysis may have been too underpowered to detect significance despite clinically meaningful trends. To further evaluate the robustness of our findings, we conducted post hoc effect size and power analyses using non-parametric tests.

The Excessive FFM Loss group demonstrated persistently elevated %FFML/BWL across all time points, with no evidence of recovery or normalization. In contrast, the control group showed a gradual increase in the %FFML/BWL over time, possibly reflecting the expected physiological adaptation. These findings suggest that patients who experience early excessive lean mass depletion after surgery may experience a chronic compositional imbalance, leading to a higher risk of developing SO. Resistance training combined with protein supplementation has shown promising outcomes in reversing lean mass reduction and increasing resting energy expenditure in long-term post-MBS patients [34]. A recent systematic review also supported the implementation of tailored exercise interventions in the early postoperative period as an effective method of preserving FFM and preventing sarcopenia [41].

Patients with lower preoperative Tyr may benefit from early, targeted interventions, such as individualized dietary counseling that emphasizes adequate protein intake (≥1.2 g/kg/day), structured exercise and resistance training, and encouragement of consistent clinic visits for the first 3 months post-surgery (the critical period for FFM loss), as well as long-term surveillance. Although formal implementation strategies remain unestablished, our findings support the integration of metabolic profiling into routine perioperative assessment protocols for MBS candidates with elevated sarcopenic risk.

### Limitations

This study has several limitations that must be acknowledged. First, the retrospective design and single-center patient cohort may have limited the generalizability of the findings. While we observed statistically significant associations, the small sample size restricted the statistical power, particularly for subgroup analyses and multivariate model robustness. With regard to the potential concerns about the modest sample size and statistical power, effect size (r) and post hoc power analyses were performed to provide a more nuanced understanding of the results. Analyses for non-parametric tests revealed that, while within-group comparisons (Wilcoxon signed-rank test) had excellent power even for medium effects (power > 0.90), between-group comparisons (Mann–Whitney U test) were underpowered for detecting medium effects (power = 0.430 at r = 0.5) (Table S5). These findings suggest the potential for false negatives in group comparisons, which should be interpreted cautiously.

In addition, while this study captured the 12-month postoperative period during which the majority of fat and lean mass changes occur, it remains unclear whether the observed early FFM loss persists, stabilizes, or recovers in the long term. Future studies need to adopt a multicenter, prospective design with a larger, more diverse population and a longer follow-up period.

Another important limitation is the method and device used for body composition measurements. In this study, BIA was the main device used for anthropometric data measurement because of its cost-effectiveness, ease of use, and low risk of radiation exposure, which allows for repeated measurements [42]. Although studies comparing BIA and dual-energy X-ray absorptiometry (DEXA) for body composition analysis have reported minimal differences between the two methods, DEXA has proven to be more accurate at the molecular level [43,44]. The relatively low precision of BIA may contribute to measurement variability or under- or overestimation of FFM, and future studies should consider utilizing DEXA to enhance data reliability.

Additionally, the lack of data on physical activity and diet pre- and postoperatively restricts the interpretation of lean mass management. This is particularly important because numerous studies have underscored the roles of exercise and diet management. A systematic review and meta-analysis on the effectiveness of exercise training after MBS demonstrated that exercise interventions, especially within 3 months after surgery, optimized BW and FM loss and improved physical fitness [45]. Nutritional strategies have shown promising roles in mitigating FFM loss in patients post-MBS. Dietary modifications such as high-protein diets or supplements exceeding 60 g/day are associated with the preservation of lean mass [46]. Patients who received daily whey protein of 30 g for 4 weeks after bariatric surgery showed increased fat loss and improved preservation of FFM and MM [47]. Accordingly, clinical or functional endpoints related to FFM, such as walking speed, resting metabolic rate, and grip strength, were not assessed; thus, the translational relevance of FFM loss cannot be directly inferred [48,49,50]. Recognizing this limitation, future studies should collect and incorporate data on diet, exercise, and clinical FFM assessment tools to further contextualize metabolic outcomes.

Despite these limitations, this study provides novel insights by identifying preoperative tyrosine levels as predictive biomarkers for patients at risk of excessive FFM loss after MBS. The findings of this study support a shift in focus from the quantity to the quality of weight loss, emphasizing the importance of preserving lean mass for homeostasis and long-term positive outcomes. Larger prospective studies with extended follow-up are needed to validate these results and optimize surgical outcomes.

## 5. Conclusions

This study suggests that preoperative AAM profiling, particularly involving tyrosine, may serve as a useful predictive tool for identifying patients at risk for excessive fat-free mass loss after metabolic and bariatric surgery. Early detection of such risks allows for individualized strategies to minimize long-term adverse outcomes. Validation in larger, diverse populations with extended follow-up and incorporation of functional FFM outcomes is essential. As obesity continues to be a global health issue, implementation strategies worldwide involve various dietary patterns, cultural differences, surgical protocols, and body composition baselines across diverse populations. This study is the first to demonstrate that preoperative tyrosine profiling may serve as an early biomarker for excessive FFM loss at postoperative 3 months following laparoscopic sleeve gastrectomy, offering opportunities for early identification and targeted interventions.

## Figures and Tables

**Table 1:** Preoperative patient demographic and anthropometric data.

	Preoperative Values (*n* = 40)
Age (years)	37.4 ± 11.3
Sex	
Male (*n*, %)	13 (32.5)
Female (*n*, %)	27 (67.5)
Concurrent MD (*n*, %)	29 (72.5)
DM (*n*, %)	16 (40%)
HTN (*n*, %)	24 (60%)
DL (*n*, %)	16 (40%)
BMI (kg/m^2^)	40.81 ± 6.37
BW (kg)	112.4 ± 21.4
FM (kg)	51.5 ± 16.0
MM (kg)	32.6 ± 6.00
FFM (kg)	61.0 ± 11.9
FFM/BW (%)	54.7 ± 8.3
%FFML/BWL	37.6 ± 28.8

Values are presented as mean ± standard deviation or number (%). Abbreviations: MD, metabolic disease; DM, type 2 diabetes mellitus; HTN, hypertension; DL, dyslipidemia; BMI, body mass index; BW, body weight; FM, fat mass; MM, muscle mass; FFM, fat-free mass; %FFML/BWL, percentage of fat-free mass loss to body weight loss.

**Table 2:** Anthropometric parameters and weight loss observed 3, 6, and 12 months postoperatively.

	Preoperative (*n* = 40)	Postoperative			*p*-Value
		3 m (*n* = 40)	6 m (*n* = 40)	12 m (*n* = 32)	
BMI (kg/m^2^)	40.8 ± 6.4	33.5 ± 5.8	30.5± 5.2	29.1 ± 5.1	<0.001 **
BW (kg)	112.4 ± 21.4	92.3 ± 18.9	84.0 ± 17.0	80.3 ± 16.9	<0.001 **
FM (kg)	51.5 ± 16.0	38.5 ± 14.0	31.8 ± 12.4	28.1 ± 10.1	<0.001 **
MM (kg)	32.6 ± 6.0	29.5 ± 5.6	28.1 ± 5.6	27.7 ± 5.6	<0.001 **
FFM (kg)	61.0 ± 11.9	53.3 ± 10.7	52.6 ± 9.6	50.9 ± 9.4	<0.001 **
FFM/BW (%)	54.7 ± 8.2	58.4 ± 8.4	63.3 ± 8.2	64.9 ± 7.9	≤0.001 **
%TWL	-	18.0 ± 5.0	25.2 ± 6.5	28.2 ± 8.2	<0.001 **
%EWL	-	40.1 ± 14.1	55.8 ± 17.2	69.2 ± 35.8	<0.001 **
%FFML/BWL	-	37.6 ± 28.8	30.0 ± 22.2	33.3± 22.9	^a^ 0.013 * ^b^ 0.455 ^c^ 0.188

Values are presented as mean ± standard deviation or number (%). *p*-values were calculated using Student’s *t*-test or the Mann–Whitney U test. * *p* < 0.05, ** *p* < 0.01. Abbreviations: BMI, body mass index; BW, body weight; FM, fat mass; MM, muscle mass; FFM, fat-free mass; %TWL, percent total weight loss; %EWL, percent excessive weight loss; %FFML/BWL, percentage of fat-free mass loss to body weight loss. ^a^ *p*-value for comparison between 3 and 6 months; ^b^ between 3 and 12 months; ^c^ between 6 and 12 months.

**Table 3:** Preoperative characteristics and anthropometric parameters of the control and Excessive FFM Loss groups.

	Control (*n* = 15)	Excessive FFM Loss (*n* = 25)	*p*-Value
Age (years)	40.3 ± 10.7	35.7 ± 11.5	0.280
Sex			0.029 *
Male (*n*, %)	8 (53.3)	5 (20.0)	
Female (*n*, %)	7 (46.7)	20 (80.0)	
Concurrent MD (*n*, %)	11 (73.3)	18 (72.0)	0.927
DM (*n*, %)	7 (46.7)	9 (36.0)	0.527
HTN (*n*, %)	10 (66.7)	14 (56.0)	0.740
DL (*n*, %)	8 (53.3)	8 (32.0)	0.205
BMI (kg/m^2^)	33.4 ± 5.0	33.6 ± 6.3	0.956
BW (kg)	92.7 ± 16.5	92.0 ± 20.5	0.783
FM (kg)	35.2 ± 9.9	40.5 ± 15.8	0.376
MM (kg)	30.4 ± 5.8	29.0 ± 5.5	0.543
FFM (kg)	57.5 ± 10.5	50.8 ± 10.2	0.106
FFM/BW (%)	66.1 ± 8.7	64.1 ± 7.4	0.505

Values are presented as mean ± standard deviation or number (%). *p*-values were calculated using the chi-square test, Student’s *t*-test, or Mann–Whitney U test. * *p* < 0.05. Abbreviations: MD, metabolic disease; DM, type 2 diabetes mellitus; HTN, hypertension; DL, dyslipidemia; BMI, body mass index; BW, body weight; FM, fat mass; MM, muscle mass; FFM, fat-free mass.

**Table 4:** %FFML/BWL, %TWL, and %EWL at postoperative 3, 6, and 12 months in the control and Excessive FFM Loss groups.

	Control (*n* = 15)	Excessive FFM Loss (*n* = 25)	*p*-Value
%FFML/BWL			
3 m	12.9 ± 12.2	52.5 ± 25.5	<0.001 **
6 m	16.8 ± 12.5	38.4 ± 22.8	<0.001 **
12 m	25.7 ± 17.1	38.5 ± 25.2	0.040 *
%TWL			
3 m	17.6 ± 5.1	18.2 ± 5.2	0.740
6 m	25.3 ± 6.5	25.2 ± 6.7	0.946
12 m	27.4 ± 8.3	28.7 ± 8.3	0.788
%EWL			
3 m	39.9 ± 14.1	40.3 ± 14.3	0.938
6 m	56.5 ± 15.9	55.3 ± 18.2	0.834
12 m	60.8 ± 18.2	74.2 ± 42.6	0.258

Values are presented as mean ± standard deviation or number (%). *p*-values were calculated using Student’s *t*-test or the Mann–Whitney U test. * *p* < 0.05, ** *p* < 0.01. Abbreviations: %FFML/BWL, percent fat-free mass loss to body weight loss; %TWL, percent total weight loss; %EWL, percent excess weight loss.

**Table 5:** Preoperative AAM values in the control and Excessive FFM Loss groups.

	Control (*n* = 15)	Excessive FFM Loss (*n* = 25)	*p*-Value
Phe	68.9 ± 11.8	68.69 ± 10.6	0.761
Trp	59.6 ± 9.0	52.30 ± 9.6	0.015 *
Tyr	64.2 ± 12.6	54.17 ± 11.5	0.025 *
5-HT	0.186 ± 0.157	0.279 ± 0.228	0.182
5-HTP	0.018 ± 0.008	0.0176 ± 0.006	0.804
5-HIAA	0.040 ± 0.013	0.0384 ± 0.018	0.406
L-DOPA	0.644 ± 0.302	0.515 ± 0.251	0.112

Values are presented as mean ± standard deviation or number (%). *p*-values were calculated using Student’s *t*-test or the Mann–Whitney U test. * *p* < 0.05. Abbreviations: Phe, phenylalanine; Trp, tryptophan; Tyr, tyrosine; 5-HT, serotonin; 5-hydroxy5-HTP, 5-hydroxytryptophan; 5-HIAA, 5-hydroxyindoleacetic acid; L-DOPA, levodopa.

**Table 6:** Logistic regression model of excessive FFM loss 3 months postoperatively: odds ratio estimates and 95% confidence intervals.

Model 1				
Step 1	*B* (s.e.)	*p*-value	Odds Ratio	95% CI
Trp	−0.084 (0.040)	0.033 *	0.919	0.850–0.993
Nagelkerke R^2^ = 0.173, *p* = 0.020 *, HL = 0.766, −2LL = 47.487				
Tyr	−0.071 (0.031)	0.022 *	0.932	0.877–0.990
Nagelkerke R^2^ = 0.528, *p* = 0.012 *, HL = 0.920, −2LL = 46.633				
**Model 2**				
Step 1	*B* (s.e.)	*p*-value	Odds Ratio	95% CI
Trp	−0.090 (0.037)	0.027 *	0.914	0.845–0.990
Age	−0.049 (0.037)	0.189	0.952	0.885–1.024
BMI	−0.013 (0.063)	0.833	0.987	0.873–1.116
FFM	−0.011 (0.035)	0.744	0.989	0.923–1.059
Nagelkerke R^2^ = 0.229, *p* = 0.118, HL = 0.538, −2LL = 45.557				
Step 1	*B* (s.e.)	*p*-value	Odds Ratio	95% CI
Tyr	−0.076 (0.032)	0.018 *	0.927	0.870–0.987
Age	−0.051 (0.038)	0.180	0.950	0.882–1.024
BMI	−0.007 (0.062)	0.915	0.993	0.879–1.122
FFM	−0.010 (0.036)	0.772	0.990	0.922–1.062
Nagelkerke R^2^ = 0.258, *p* = 0.079, HL = 0.721, −2LL = 44.545				
**Model 3**				
Step 1	*B* (s.e.)	*p*-value	Odds Ratio	95% CI
Trp	−0.071 (0.042)	0.093	0.931	0.857–1.012
Sex (Male)	−2.748 (1.261)	0.029 *	0.064	0.005–0.758
Age	−0.030 (0.040)	0.452	0.970	0.897–1.049
BMI	0.010 (0.068)	0.882	1.010	0.884–1.154
FFM	0.061 (0.052)	0.234	1.063	0.961–1.177
Nagelkerke R^2^ = 0.390, *p* = 0.019 **, HL = 0.434, −2LL = 39.422				
Step 1	*B* (s.e.)	*p*-value	Odds Ratio	95% CI
Tyr	−0.105 (0.041)	0.012 *	0.901	0.830–0.977
Sex (Male)	−3.990 (1.513)	0.008 **	0.018	0.001–0.359
Age	−0.029 (0.043)	0.508	0.972	0.893–1.058
BMI	0.021 (0.072)	0.768	1.022	0.886–1.177
FFM	0.083 (0.053)	0.120	1.086	0.979–1.206
Nagelkerke R^2^ = 0.528, *p* = 0.001 **, HL = 0.502, −2LL = 33.335				

B: Beta coefficient (magnitude indicates the strength of the relationship; >0, positive correlation; < 0, negative correlation). s.e.: standard error (variability of B; smaller s.e.: greater precision). Nagelkerke R^2^: fitness of the model (a higher value indicates a better model). HL: Hosmer–Lemeshow *p*-value (*p* > 0.05, acceptable model fit; i.e., predicted probabilities aligned with observed outcomes). –2LL: −2 Log Likelihood (smaller values indicate better model fit). * *p* < 0.05, ** *p* < 0.01. Abbreviations: Trp, tryptophan; Tyr, tyrosine; BMI, body mass index; FFM, fat-free mass.

**Table 7:** ROC curve analysis of preoperative Tyr levels.

	AUC ^a^	SE ^b^	95% Confidence Interval ^c^		*p*-Value ^d^	Cut-Off ^e^
			Lower	Upper		
Tyr	0.715	0.084	0.550	0.880	0.025 *	≥54.82

^a^ AUC: area under the receiver operating characteristic curve. ^b^ SE: standard error. ^c^ CI: confidence interval for the AUC. ^d^ *p*-value testing whether the AUC is significantly different from 0.5. * *p* < 0.05. **e** Cut-off value derived from Youden’s index for optimal sensitivity and specificity. Note: only the ROC curve for Tyr is presented. Other variables were not plotted due to lack of statistical significance.

## Data Availability

The data presented in this study are available upon request from the corresponding author owing to privacy restrictions.

## Supplementary Materials

The following supporting information can be downloaded at: https://www.mdpi.com/article/10.3390/metabo15080543/s1, Table S1. Wilcoxon signed-rank test of Table 2 variables comparing pre- and postoperative values across multiple timepoints; Table S2. Mann-Whitney U test of Table 3 baseline demographic and body composition variables between two groups; Table S3. Mann-Whitney U test of Table 4 variables comparing weight loss and body composition indicators at postoperative 3, 6, and 12 months between the two groups; Table S4. Mann-Whitney U test of Table 5 preoperative metabolite profiles; Table S5. Post hoc power analysis for Mann-Whitney U and Wilcoxon signed-rank tests conducted using G*Power (ver 3.1.9.7), based on assumed effect sizes (r = 0.3, 0.5, 0.7). Sufficient power was achieved only for Wilcoxon tests with medium or large effects; Table S6. Tolerance and variance inflation factor (VIF) values for covariates in Table 6, demonstrating absence of multicollinearity.

## Abbreviations

The following abbreviations are used in this manuscript:

FFM	Fat-free mass
BW	Body weight
MBS	Metabolic and bariatric surgery
BMI	Body mass index
AAMs	Amino acid metabolites
%FFML/BWL	Percent of fat-free mass loss relative to body weight loss
%TWL	Percent total weight loss
%EWL	Percent excess weight loss
ROC	Receiver operating characteristic
FM	Fat mass
Tyr	Tyrosine
Trp	Tryptophan
