# Supplementary material for: Preoperative Tyrosine Levels as Predictive Biomarkers for Excessive Fat-Free Mass Loss Following Laparoscopic Sleeve Gastrectomy in Patients with Morbid Obesity
Source: Metabolites. 2025 Aug 11;15(8):543. doi: 10.3390/metabo15080543 (PMC12388380; doi:10.3390/metabo15080543)
Supplement: Supplementary file 1 [file metabolites-15-00543-s001.zip › metabolites-3758785-supplementary.pdf]

Supplementary Table S1. Wilcoxon signed-rank test of Table 2 variables comparing pre- and postoperative values across multiple timepoints.

Variable	Timepoint	N	Z	r (effect size)
BMI	Preop vs. Postop 3 mo	40	-5.511	-.871
	Preop vs. Postop 6 mo	40	-5.511	-.871
	Preop vs. Postop 12 mo	38	-5.373	-.872
BW	Preop vs. Postop 3 mo	40	-5.511	-.871
	Preop vs. Postop 6 mo	40	-5.511	-.871
	Preop vs. Postop 12 mo	38	-5.373	-.872
FM	Preop vs. Postop 3 mo	40	-5.229	-.827
	Preop vs. Postop 6 mo	40	-5.497	-.869
	Preop vs. Postop 12 mo	32	-4.937	-.873
MM	Preop vs. Postop 3 mo	40	-4.713	-.745
	Preop vs. Postop 6 mo	40	-5.377	.-850
	Preop vs. Postop 12 mo	32	-4.815	-.851
FFM	Preop vs. Postop 3 mo	40	-5.336	-.844
	Preop vs. Postop 6 mo	40	-5.404	-.854
	Preop vs. Postop 12 mo	31	-4.860	-.873
FFM/BW	Preop vs. Postop 3 mo	40	-3.972	-.628
	Preop vs. Postop 6 mo	40	-5.067	-.801
	Preop vs. Postop 12 mo	32	-4.394	-.777
%TWL	Postop 3 mo vs. Postop 6 mo	40	-5.511	-.871
	Postop 3 mo vs. Postop 12 mo	38	-5.373	-.872
	Postop 6 mo vs. Postop 12 mo	38	-4.458	-.723
%EWL	Postop 3 mo vs. Postop 6 mo	40	-5.511	-.871
	Postop 3 mo vs. Postop 12 mo	40	-5.511	-.871
	Postop 6 mo vs. Postop 12 mo	40	-4.689	-.741
%FFML/BWL	Postop 3 mo vs. Postop 6 mo	40	-2.850	-.451*
	Postop 3 mo vs. Postop 12 mo	32	-1.215	-.215*
	Postop 6 mo vs. Postop 12 mo	32	-.926	-.164*

Effect size calculated as Z/√N. According to Cohen's (1988) criteria, effect sizes are interpreted as follows: r < 0.1 = negligible, 0.1-0.3 = small, 0.3-0.5 = medium, and ≥ 0.5 = large; all values not meeting the threshold for a large effect size are marked with *. BMI, body mass index; BW, body weight; FM, fat mass; MM, muscle mass; FFM, fat-free mass; %TWL, percent total weight loss; %EWL, percent excessive weight loss; %FFML/BWL, percent of fat-free mass loss to body weight loss; Preop, preoperative; Postop, postoperative; mo, months.

Supplementary Table S2. Mann-Whitney U test of Table 3 of baseline demographic and body composition variables between two groups.

Variable	N	Z	r (effect size)
Age	40	-1.105	-.175
Concurrent MD	40	-.090	-.014*
DM	40	-.658	-.104*
HTN	40	-.658	-.104*
DL	40	-1.317	-.208*
BMI	40	-.182	-.029*
BW	40	-.237	-.037*
FM	40	-.601	-.095*
MM	40	-.587	-.093*
FFM	40	-.196	-.031*
FFM/BW	40	-.084	-.013*

According to Cohen's (1988) criteria, effect sizes are interpreted as follows: r < 0.1 = negligible, 0.1-0.3 = small, 0.3-0.5 = medium, and ≥ 0.5 = large; all values not meeting the threshold for a large effect size are marked with *. MD, medical disease; DM, type 2 diabetes mellitus; HTN, hypertension; DL, dyslipidemia; BMI, body mass index; BW, body weight; FM, fat mass; MM, muscle mass; FFM, fat-free mass.

Most variables demonstrated negligible to small effect sizes.

Supplementary Table S3. Mann-Whitney U test of Table 4 of variables comparing weight loss and body composition indicators at postoperative 3, 6, and 12 months between the two groups.

Variable	Timepoint	N	Z	r (effect size)
%FFML/BWL	Postop 3 mo	40	-5.238	-.828
	Postop 6 mo	40	-3.478	-.550
	Postop 12 mo	32	-2.053	-.363*
%TWL	Postop 3 mo	40	-.489	-.077*
	Postop 6 mo	40	-.154	-.024*
	Postop 12 mo	38	-.269	-.044*
%EWL	Postop 3 mo	40	-.070	-.011*
	Postop 6 mo	40	-.405	-.064*
	Postop 12 mo	40	-.485	-.077*

According to Cohen's (1988) criteria, effect sizes are interpreted as follows: $r < 0.1$ = negligible, 0.1-0.3 = small, 0.3-0.5 = medium, and ≥ 0.5 = large; all values not meeting the threshold for a large effect size are marked with *. %FFML/BWL, percent of fat-free mass loss to body weight loss; %TWL, percent total weight loss; %EWL, percent excessive weight loss; Preop, preoperative; Postop, postoperative; mo, months.

Notably, %FFML/BWL at postoperative 3 and 6 months demonstrated moderate to large effect sizes.

Supplementary Table S4. Mann-Whitney U test of Table 5 of preoperative metabolite profiles.

Variable	N	Z	r (effect size)
Phe	40	-.321	-.051*
Trp	40	-2.445	-.387*
Tyr	40	-2.249	-.356*
5-HT	40	-1.355	-.214*
5-HTP	40	-.266	-.042*
5-HIAA	40	-.839	-.133*
L-DOPA	40	-1.529	-.242*

According to Cohen's (1988) criteria, effect sizes are interpreted as follows: $r < 0.1$ = negligible, 0.1-0.3 = small, 0.3-0.5 = medium, and ≥ 0.5 = large; all values not meeting the threshold for a large effect size are marked with *. Phe, phenylalanine; Trp, tryptophan; Tyr, tyrosine; 5-HT, serotonin; 5-hydroxy5-HTP, 5-hydroxytryptophan; 5-HIAA, 5-hydroxyindoleacetic acid; L-DOPA, levodopa.

Trp and Tyr demonstrated moderate effect sizes, consistent with their predictive roles.

Supplementary Table S5. Post-hoc power analysis for Mann-Whitney U and Wilcoxon signed-rank tests conducted using G*Power (ver 3.1.9.7), based on assumed effect sizes (r = 0.3, 0.5, 0.7). Sufficient power was achieved only for Wilcoxon tests with medium or large effects.

**Analysis Type**	**r (effect size)**	**Power**
Mann-Whitney U	0.3	.222
Mann-Whitney U	0.5	.430
Mann-Whitney U	0.7	.659
Wilcoxon Signed Rank	0.3	.570
Wilcoxon Signed Rank	0.5	.917
Wilcoxon Signed Rank	0.7	.995

Statistical power is generally interpreted as follows: ≥ 0.80 = sufficient; 0.50-0.79 = moderate; and < 0.50 = low power.

Wilcoxon signed-rank tests demonstrated high post-hoc statistical power, with power estimates of 0.917 for medium effect size (r = 0.5) and 0.995 for large effect size (r = 0.7), indicating sufficient sample size for detecting clinically meaningful effects.

Supplementary Table S6. Tolerance and variance inflation factor (VIF) values for covariates in Table 6, demonstrating absence of multicollinearity.

Variable	Tolerance	VIF
Trp	.555	1.803
Tyr	.586	1.706
Sex	.569	1.759
Age	.774	1.291
BMI	.845	1.184
FFM	.479	2.089

VIF, variance inflation factor; Trp, tryptophan; Tyr, tyrosine; BMI, body mass index; FFM, fat-free mass.

In this study, all tolerance values were above 0.1 (minimum = 0.479), and all VIF values were below 5 (maximum = 2.089), indicating no concerns for multicollinearity. Therefore, the likelihood of coefficient instability or statistical distortion due to excessive correlation among independent variables is low, and the regression analysis results can be considered reliable.
